# Prevalence of Iron Deficiency Anemia in Biochemically Defined Moderate to Severely Anemic patients in a Tertiary Care Centre

**DOI:** 10.31729/jnma.4632

**Published:** 2019-10-31

**Authors:** Niharika Shah, Sairil Pokharel, Deebya Raj Mishra, Purbesh Adhikari

**Affiliations:** 1 Department of Pathology, B. P. Koirala Institute of Health Sciences, Dharan, Nepal; 2 Department of Internal Medicine, B. P. Koirala Institute of Health Sciences, , Dharan, Nepal

**Keywords:** *bone marrow iron*, *intensive method*, *serum ferritin*

## Abstract

**Introduction:**

Anemia due to iron deficiency and chronic diseases is a common occurrence in a developing country like Nepal, the latter seen in patients with various inflammatory, autoimmune, and malignant disorders (functional iron deficiency). The Intensive method of marrow iron examination, which this study has employed, provides a clinically useful iron status classification in cases of functional iron deficiency. The aim of the study is find out the prevalence of iron deficiency anemia in biochemically defined moderate to severe anemic patients in tertiary care center.

**Methods:**

A descriptive cross-sectional study was done in 43 patients who underwent bone marrow aspiration for evaluation of any cause and had moderate to severe anemia at the same time over a period of one year from Nov 2015 to 2016. Ethical clearance was obtained from Institutional Review Committee. The bone marrow iron stores were assessed by the “intensive method” apart from the routinely used Gale’s method. Data was collected and entry were done in Statistical Package for Social Sciences version 24. Point estimate at 95% Confidence Interval was calculated along with frequency and proportion for binary data.

**Results:**

The intensive grading system demonstrated normal marrow iron store in 13 (30.2%), depleted iron stores in 3 (7%), functional iron deficiency in 14 (32.6%), and combined deficiency in 13 (30.2%) patients. Mean log ferritin concentration was lower in patients with depleted iron stores (2.2µg/l) than in those with normal (2.7µg/l), and functional iron deficiency (2.4µg/l). The mean log ferritin in combined deficiency was lower than the mean log ferritin concentration in iron store defficiency (1.9µg/l).

**Conclusions:**

The prevalence of functional iron deficiency anemia was greatest when the intensive method for assessment of bone marrow iron was used, thus differentiating four different iron status categories, including functional iron deficiency, from actual iron store deficiency, avoiding unnecessary iron supplementation in the former group.

## INTRODUCTION

Anemia due to iron deficiency and chronic diseases is a common occurrence in a developing country like Nepal, the latter seen in patients with various inflammatory, autoimmune, and malignant disorders (functional iron deficiency). Bone marrow microscopy (Gale’s method, evaluating hemosiderin content within fragments) has been the gold standard for assessing iron deficiency.^1-3^ Individual macrophages, however can be observed for iron (indicating iron depletion)^4^ and decrease in erythroblast iron may indicate functional iron deficiency.^5^ Iron deficiency anemia and anemia of chronic disease are quite common in Nepal. Thus a newer intensive bone marrow iron grading^4^ can be used to assess iron in fragments, macrophages and erythroblasts, facilitating distinction of functional) anemia of chronic diseases, (from iron store deficiency, preventing unnecessary iron supplementation in the former.

The aim of the study is find out the prevalence of iron deficiency anemia in biochemically defined moderate to severe anemic patients in tertiary care center.

## METHODS

This descriptive cross-sectional study was conducted on 43 patients admitted with moderate to severe anemia, in whom a diagnostic bone marrow examination was requested by the clinician for some other purpose at a tertiary care hospital from November 2015 to November 2016.

Sample size estimation:

n = Z^2^ × p × q/e^2^

    = 43

where,

n = sample size for infinite population
Z = 1.96 at 95% Confidence Interval
p = prevalence of iron deficiency anemia in biochemically defined moderate to severely anemic patient= 10% (educated guess)
q = 1- P
e = margin of error= 9%

A purposive sampling method was used. The inclusion criteria for the study being patients admitted in the department of Internal Medicine with moderate to severe anemia (with or without other comorbidities), with a bone marrow aspiration performed and evaluation of serum ferritin level done. The exclusion criteria were patients who did not give consent for the study, patients who did not have anemia and when an unsatisfactory bone marrow aspiration sample was received. This study was granted ethical approval by the Institutional Review Committee (IRC), BPKIHS, Dharan, Nepal.

Written informed consent was taken and a bone marrow aspirate was obtained from the posterior superior iliac spine with all aseptic precautions, spread onto a slide, air dried, fixed with methanol, and stained with Perl’s Prussian blue stain. A positive control was included in each batch of slides. Marrow smears with at least seven fragments were subjected for microscopic examination.^6^ Peripheral venous blood was also collected for hemoglobin and serum ferritin level estimation.

The Gale’s grading method,^3^ which is routinely used method was first employed to assess iron in the marrow fragment, grades 0 and 1 corresponding to none and very slight marrow iron, respectively, were considered indicative of iron store deficiency.

**Figure 1:**
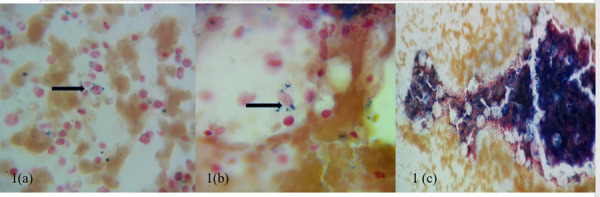
a) Iron granules in erythroblast cytoplasm (arrow); (Perl’s Prussian blue stain x1000), (b) Iron granules within a macrophage cytoplasm (arrow); (Perl’s Prussian blue stain x1000), (c) Marrow with increased iron stores, Gale’s grade 5 (Perl’s Prussian blue stain x 100).

Next, the intensive method of assessing marrow iron in three sites, namely in the marrow fragments, macrophages around the marrow fragments, and erythroblasts was performed.7 The marrow fragment iron was assessed and then, under oil immersion, 20 fields around the fragments were further examined for the presence of macrophages with iron and 100 erythroblasts were examined for iron and the percentage of sideroblasts (erythroblasts containing iron granules in their cytoplasm) noted (Figure 1). When <30% of erythroblasts had iron granules, it was considered to be erythroblast iron deficiency^4^ (Figure 1).

Iron status assessed by the intensive method was categorized as normal, functional iron deficiency, iron stores deficiency, or combined functional iron and iron stores deficiency.

Bone marrow iron results were compared with serum ferritin level. Hemoglobin was estimated using the automated cell counter, Lablife.

Data was collected and entry were done in SPSS version 24. Point estimate at 95% Confidence Interval was calculated along with frequency and proportion for binary data.

## RESULTS

The iron status category of the 43 patients assessed by both the intensive method as well as the conventional Gale’s method (Table 1). The intensive grading system demonstrated normal marrow iron stores in 13 (30.2%) of cases, depleted iron stores in 3 (7.0%), functional iron deficiency in 14 (32.6%) in 95% C.I [18.61%-46.59%], and combined deficiency was detected in 13 (30.2%) of patients, thus distinguishing iron store deficiency from functional iron deficiency states.

Of the 43 patients recruited in the study, 23 (53.5%) were male and 20 (46.5%) were female. Socioeconomic status of most of these patients was middle class, 18 (41.9%), followed by lower class, 14 (32.6%). None belonged to upper class. Most of the patients presented with fever, 12 (27.9%), followed by fatigue, shortness of breath, and other causes. Eventual bone marrow diagnosis in most of these patients were non-specific, followed by megaloblastic anemia and mixed nutritional anemia. The minimum age was 8 years and maximum 85 years (42.3±23.8). 31 patients (72.1%) patients presented with severe anemia (hemoglobin <7 g/dl), whereas 12 (27.9%) patients had moderate anemia (hemoglobin 7-10 g/dl). The minimum hemoglobin noted was 2.6 g/dl and maximum 9.5 g/dl (mean 5.9±2.1g/dl).

The Gale’s grading method revealed hypo-ferremic state in 16 (37.2%) cases, normal iron stores in 10 (23.3%) cases, slightly increased in 6 (14%), moderately increased in 5 (11.6%), and markedly increased in 6 (4%).

**Table 1:** Results of Bone marrow iron status category (n=43).

Method	Iron status category	n (%)
Gale’s method	Iron deficiency	16 (37.2)
	Normal	10 (23.3)
	Slightly increased	6 (14)
	Moderately increased	5 (11.6)
	Markedly increased	6 (4)
Intensive method	Normal	13 (30.2)
	Functional iron deficiency	14 (32.6)
	Iron store deficiency	3 (7.0)
	Functional and iron store deficiency	13(30.2)

**Figure 2:**
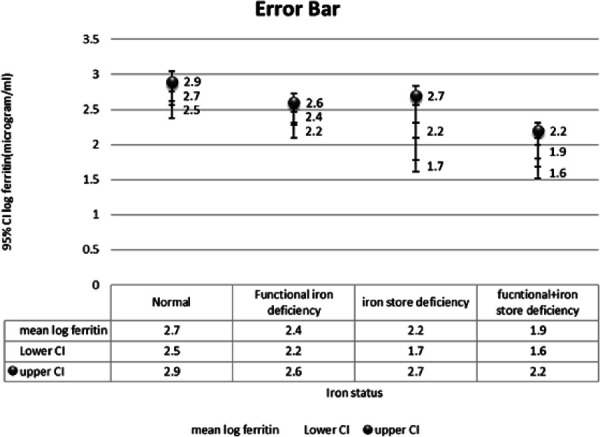
Mean log ferritin for the different iron status categories using the intensive grading method represented in an Error bar graph.

## DISCUSSION

The intensive histological grading method of assessing bone marrow iron stores is different from the traditional Gale’s method in that it differentiates four different iron states instead of two categories in Gale’s method.^4^ The intensive method attempts to distinguish functional iron deficiency state, which is the inadequate iron delivery to the erythroblasts despite having an adequate fragment iron from actual iron store deficiency where iron is deficient in fragments as well. Functional iron deficiency is usually seen in anemia of chronic diseases. It is important to distinguish between the two to avoid unnecessary iron supplementation in anemia of chronic diseases.^8^

The conventional method of assessing the body iron reserve is by assessing the biochemical markers of iron metabolism, one of which being serum ferritin. Ferritin, however is also an acute phase response protein, and thus may not be a very reliable marker when infection and inflammation both coexist.^9–11^

The levels of mean log ferritin were increased in all patients with functional iron deficiency 32.6% in this study (2.4 microgram/l), as compared to patients with actual iron store deficiency 7% (mean log ferritin: 2.2 microgram/l). All patients in these two subsets had severe anemia as well. This finding helps corroborate the fact that anemia in the functional iron deficiency subset was most likely due to chronic diseases and inflammation and thus classifying the iron stores according to the intensive grading method helped delineate these group of patients in whom iron treatment for anemia would not have worked. Phiri, et al. came to a similar conclusion as well, as in their study children with functional iron deficiency had significantly higher levels of CRP, an acute phase reactant thus supporting the hypothesis that these children had anemia of inflammation.^4^

However, the mean log ferritin level came out to be significantly lower in patients with combined deficiency (functional and iron store deficiency) (1.9 microgram/l) as compared to functional iron deficiency (2.4 microgram/l). This finding is contrary to the conclusion drawn by Bableshwor et al,^8^ where they found that ferritin levels may not be very useful in differentiating functional iron deficiency from functional and iron store deficiency.

This study observes a lower prevalence of iron store deficiency 7% in moderately to severely anemic patients. This could be due to the fact that in a hospital- based setting, most people undergoing a bone marrow examination were already mostly suffering from a chronic disease, and their anemia was accountable to the chronic diseases they were suffering from rather than actual iron store deficiency.

According to Gale’s method, all cases with an increased iron in bone marrow, i.e., slightly increased iron stores 14%, moderately increased iron stores 11.6%, and markedly increased iron stores 14%, fell under Functional iron deficiency status in the intensive method of assessing bone marrow iron stores thus allowing a precise iron status categorization in these patients despite an increased iron stores in the bone marrow.

There was a discrepancy however in the categorization of normal between the Gale’s method, 23.3% and the intensive method, 30.2%. This could be because any iron stores that were greater than score 2 in the Gale’s method were classified as slightly increased, moderately increased or markedly increased and not as normal, however any iron score greater than 2 were categorized as normal in the intensive method if all the other variables fell into place (iron present in erythroblast, bone marrow fragment and macrophage or iron present in bone marrow fragment and erythroblast). Thus, it could be that the Gale’s method could be a better assessor of classifying increase in iron stores whereas the intensive method failed to do so.

However, the use of erythroblast iron to delineate iron status has certain limitations. Marrow smears in our study were counterstained with neutral red giving a uniform pink background color making distinction of cell types challenging and may have led to errors in determining the actual erythroblast iron status. Other counter stains like May Grunwald Giemsa or hematoxylin have been used in other studies as a measure to improve cellular detail.^12^

## CONCLUSIONS

The prevalence of functional iron deficiency anemia was greatest when the intensive method for assessment of bone marrow iron was used. Despite some of the aforementioned shortcomings, an intensive method of assessing bone marrow iron stores may thus be of clinical significance as it differentiates four different iron status categories, mainly functional iron deficiency, seen in patients with anemia of chronic diseases from iron store deficiency seen in patients with iron deficiency anemia, therefore avoiding unnecessary iron supplementation in the former group.

## Conflict of Interest:

None.
